# SIRT1 promotes glucolipid metabolic conversion to facilitate tumor development in colorectal carcinoma

**DOI:** 10.7150/ijbs.76704

**Published:** 2023-03-27

**Authors:** Zhihao Wei, Jianyu Xia, Jiatao Li, Jingshu Cai, Juanjuan Shan, Chengcheng Zhang, Lu Zhang, Ting Wang, Cheng Qian, Limei Liu

**Affiliations:** 1 Chongqing Key Laboratory of Translational Research for Cancer Metastasis and Individualized Treatment, Chongqing University Cancer Hospital, Chongqing 400030, China.; 2 College of Basic Medical Sciences, Third Military Medical University, Chongqing 400038, China.; 3 Department of Hepatobiliary Surgery, Southwest Hospital, Third Military Medical University, Chongqing 400038, China.; 4 School of Medicine Chongqing University, Chongqing 400030, China.

**Keywords:** β-catenin, colorectal cancer, metabolic glucolipid reprogramming, sirtuin-1, tumor development.

## Abstract

**Background:** Fatty acid oxidation (FAO) is a major alternate energy metabolism pathway in tumor cells subjected to metabolic stress caused by glucose deficiency during rapid progression. However, the mechanism of metabolic reprogramming between glycolysis and FAO in tumor cells is unknown. Therefore, identifying the metabolic glucolipid conversion hub in tumor cells is crucial.

**Methods:** We used single-cell RNA sequencing (scRNA-Seq), RNA sequencing (RNA-Seq), The Cancer Genome Atlas (TCGA), and chromatin immunoprecipitation sequencing (ChIP-Seq) to predict the critical regulator and mechanism of metabolic glucolipid conversion in colorectal cancer (CRC) tumor cells. We used Seahorse metabolic analysis, immunoblotting, immunofluorescence, and immunohistochemical (IHC) technology to verify the prediction and mechanism of this regulator in cancer cell lines, a nude mouse xenograft model, and clinical CRC samples.

**Results:** We demonstrated that sirtuin-1 (SIRT1) was upregulated in CRC cells in response to glucose deprivation and oxidative stress. SIRT1 was also a hub of metabolic glucolipid conversion. SIRT1 upregulation deacetylated β-catenin, translocated it from the nucleus to the cytoplasm, attenuated glycolysis, and was positively correlated with fatty acid oxidation (FAO). Clinical analysis of SIRT1 expression in tumor tissues showed the SIRT1^High^ profile was associated with poor prognosis in CRC patients. SIRT1 interference therapy significantly suppressed tumors in the mouse xenograft model.

**Conclusions:** In hostile, glucose-deficient TMEs, SIRT1 is upregulated, and CRC cells transform the Warburg phenotype to FAO. SIRT1 indicates the frequency of glucolipid transformation and rapid tumor progression and is a promising therapeutic target of CRC.

## Introduction

In tumorigenesis, metabolic reprogramming enables cancer cells to contend with energy source limitations 1, 2. Cancer cells may undergo anaerobic glycolysis, facilitating rapid ATP synthesis and biosynthesis 3. However, rapid tumor proliferation creates a nutrient-deficient tumor microenvironment (TME), and certain malignant cells must switch to other metabolic routes to survive. Hence, regulating metabolic reprogramming during energy stress is essential for successful tumor development. Identifying the hub of this process is of great significance in metabolism-based cancer therapy.

Recent work demonstrated that lactate dehydrogenase A (LDHA) promotes and maintains the Warburg effect 4, 5. In glycolysis, LDHA converts pyruvate to lactate under the transcriptional regulation of *c-Myc* 6. The latter is well-established as a target gene of the β-catenin/TCF transcription factor (TF) complex 7. Acetylation promotes nuclear translocation and transcription of β-catenin by forming β-catenin-LEF/TCF complexes in the nucleus 8, 9. Though β-catenin induces glycolysis-targeting genes, the effects of its acetylated state on tumor cell metabolism are unknown.

Glucose and fatty acids are the most important energy sources in the TME 10. Cancer cells might resort to lipid metabolism under glucose deficiency. Recent studies have focused on fatty acid β-oxidation (FAO) in tumors. Multiple malignancies presented with significant overexpression of the FAO enzymes CPT1A, CPT1B, and CPT-2 11, 12. A prior study reported that colorectal cancer (CRC) cells facilitated FAO by radiating towards adipocyte-rich tissues and absorbing fatty acids (FAs) 13. Solid tumors generate abundant ATP during metabolic stress and utilize FAO in nicotinamide adenine dinucleotide phosphate (NADPH) production to neutralize oxidative stress 1. The dual functions of FAO are necessary to ensure cancer cell survival as reactive oxygen species (ROS) accumulation is a hallmark trait of the TME 14. An earlier investigation demonstrated that FAO favors tumor cell proliferation, survival, drug resistance, and metastatic progression. However, the mechanisms by which it promotes these processes in CRC are unknown 15.

Sirtuins are nicotinamide adenine dinucleotide (NAD^+^)-dependent histone deacetylases that regulate energy metabolism and senescence 16. They are abundant in normal tissues but relatively less common in different tumors. A prior study highlighted the tumor-promoting role of sirtuins 17. Our previous research disclosed the role of SIRT1 as a hub in metabolic reprogramming during liver cancer stem cell differentiation 18. Sirtuins directly or indirectly regulate lipid metabolism. SIRT3 and SIRT5 induce FAO by deacetylating and activating key enzymes. SIRT1 complexes with FOXO3a and NRF1 on the SIRT6 promoter and controls the transcription of genes encoding key enzymes and FA transporters 19-21. However, the roles of SIRT1 in glucolipid metabolism conversion in CRC cells are unknown.

In the present work, we established that SIRT1 is closely associated with FAO and found that CPT1A and SIRT1 were strongly upregulated under glucose--deficient conditions. In contrast, SIRT1 upregulation deacetylated β-catenin, translocated it to the cytoplasm, and attenuated glycolysis. As SIRT1 deacetylates β-catenin and facilitates FAO, it plays a pivotal role in the metabolic conversion of glycolipids in CRC cells. Furthermore, the SIRT1 expression levels in colorectal tumor tissues may reflect the frequency of transition of glycolipid metabolism and serve as a therapeutic target and promising metabolic indicator of tumor progression.

## Results

### SIRT1 is essential for CRC cells to respond to glucose deprivation

**Figure 1:**
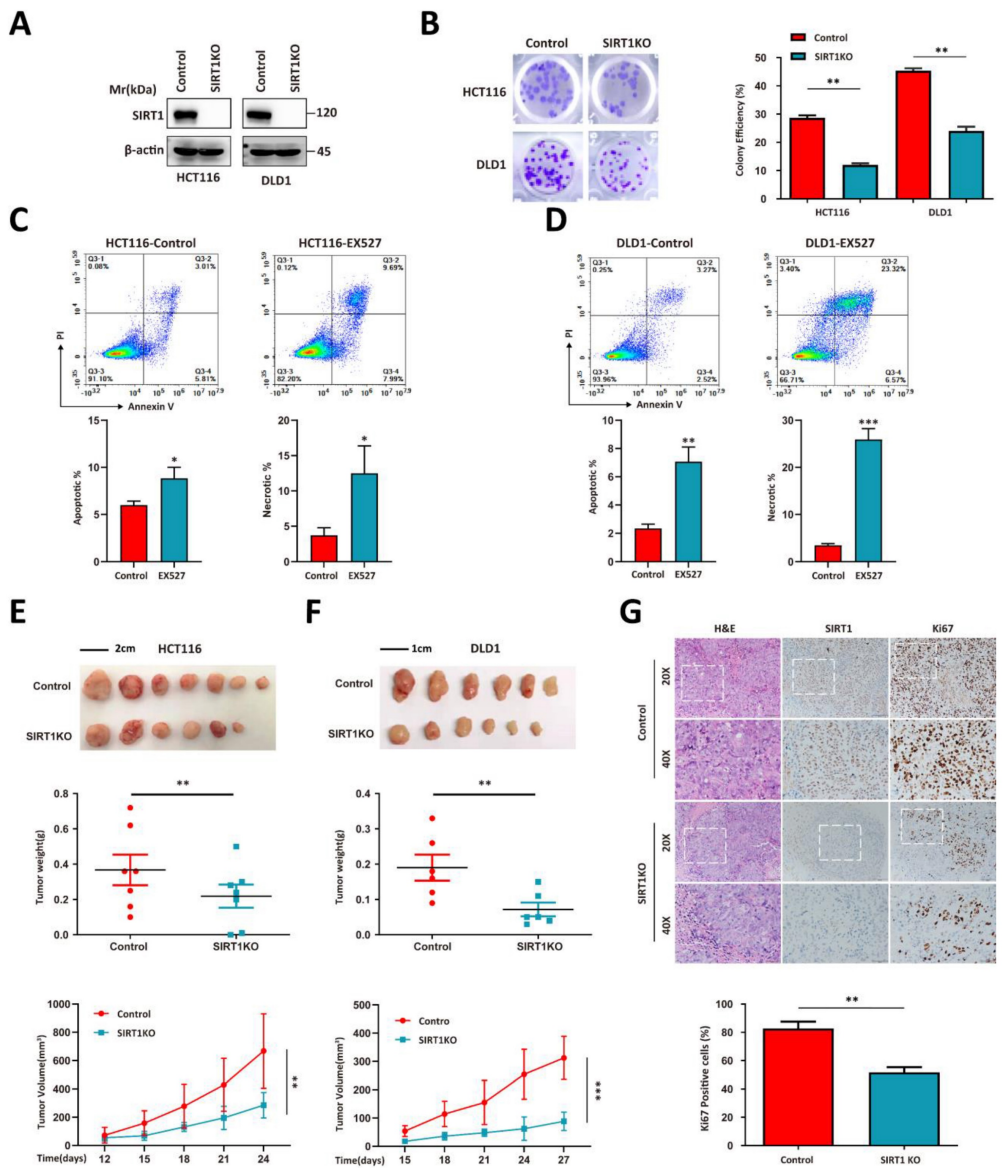
** SIRT1 determines the proliferation capacity of colorectal tumor cells. (A)** HCT116 and DLD1 cells were transfected with the CRISPR/Cas9 plasmids known to target SIRT1. SIRT1 KO was confirmed by western blotting. **(B)** Colony-forming efficiency was determined for the control and SIRT1 KO cells. The initial cell count was 50. (C-D) Apoptosis and necrosis ratios were determined for HCT116 and DLD1 cells treated either with dimethyl sulfoxide (DMSO) or 20 μM EX527. **(E)** HCT116 and SIRT1 KO HCT116 cell tumorigenicities were determined by subcutaneously injecting them (1 × 10^6^/mouse; n = 7/group) into nude mice. From day 12 after the initial injection, tumor volumes were measured with a vernier caliper every 3 d. Tumor tissues were collected and weighed at the end of the assay. **(F)** DLD1 and SIRT1 KO DLD1 cell tumorigenicities were determined by subcutaneously injecting them (1 × 10^6^/mouse, n = 6/group) into nude mice. From day 15 after the initial injection, tumor volumes were measured with a vernier caliper every 3 d. Tumor tissues were collected and weighed at the end of the assay. **(G)** Representative IHC staining of H&E, Ki-67, and cleaved caspase3 in tumor tissues (DLD1) collected at the end of the assay. Scale bars = 100 μm (left panel) and 50 μm (right panel). Cells and Ki-67-positive cells in images were enumerated with ImageJ (NIH, Bethesda, MD, USA). Ki-67-positive cell ratios were compared between control and SIRT1 KO groups. *P < 0.05, **P < 0.01, ***P < 0.001, ns: nonsignificant.

We used the HCT116 and DLD1 human colorectal tumor cell lines to perform a CRISPR/Cas9-based *SIRT1* knockout (KO) and evaluate the correlation between *SIRT1* and tumor growth (Figure 1A). SIRT1 KO attenuated the colony formation capacity (Figure 1B). The addition of the SIRT1 inhibitor EX527 into the tumor cell culture system significantly increased the apoptotic ratio (Figures 1C-1D). We then used SIRT1 KO and wild-type (WT) cells in *in vivo* assays and found that the proliferative activity of the former was inferior to those of the control groups (Figures 1E-1F). Immunohistochemical (IHC) assays of the tumor tissues revealed that SIRT1 deficiency downregulated the Ki-67 proliferation marker in tumor cells (Figure 1G). These data suggest that SIRT1 plays a vital role in CRC tumor development.

**Figure 2:**
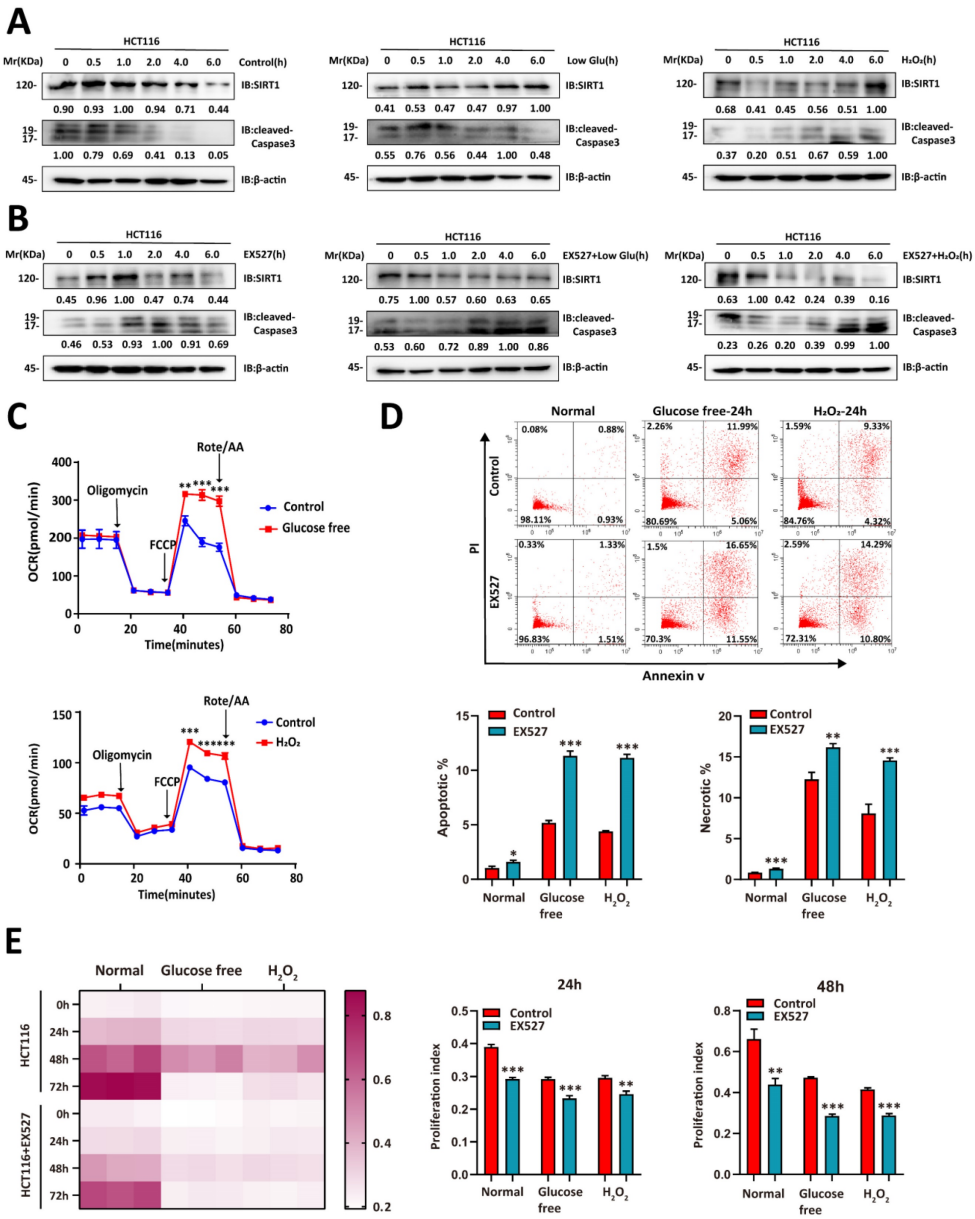
** SIRT1 enables CRC cells to contend with glucose deprivation. (A)** HCT116 cells were incubated in a glucose-free medium (DMEM plus 10% (v/v) FBS without sodium bicarbonate, glucose, *L*-glutamine, or sodium pyruvate) or treated with 1 mM H_2_O_2_. SIRT1 and cleaved caspase3 expression levels were measured by western blotting. **(B)** 20 μM EX527 was added to the culture in **(A)**. SIRT1 and cleaved caspase3 expression levels were measured by western blotting. **(C)** OCR was examined in HCT116 cells treated either with glucose-free medium or 1 mM H_2_O_2_ for 6 h. Data are means ± SEM of three independent assays. Each assay consisted of two wells per treatment and control group. **P < 0.01, ***P < 0.001. **(D)** Flow cytometry was used to detect apoptosis and necrosis rates in HCT116 and SIRT1-inhibited HCT116 cells after treatment either with glucose-free medium or 1 mM H_2_O_2_ for 24 h. Data are means ± SEM of three independent assays. *P < 0.05, **P < 0.01, ***P < 0.001. **(E)** CCK-8 assay was run to evaluate the proliferative capacities of HCT116 and SIRT1-inhibited HCT116 cells under glucose-free and 1 mM H_2_O_2_ conditions. Proliferation indices between control and EX527 treatment groups were compared at 24 h and 48 h. **P < 0.01, ***P < 0.001.

We then evaluated whether glucose deprivation affects *SIRT1* expression. Glucose deprivation impairs glycolysis, induces oxidative stress, causes redox imbalance, and reduces the energy supply 22. We cultured CRC cells in a sugar-free or H_2_O_2_-supplemented medium and observed that SIRT1 upregulation stabilized the condition of tumor cells under stress stimulus. They presented with downregulation of the apoptosis-related protein cleaved caspase3 (Figure 2A). However, SIRT1 inhibition weakened the ability of tumor cells to endure metabolic stress and upregulated cleaved caspase3 (Figure 2B). The oxygen consumption rate (OCR) was enhanced in the CRC cells under hostile environments. This response indicates oxidative metabolic reprogramming (Figure 2C).

We then explored whether SIRT1 augmented the ability of tumor cells to contend with stress conditions. Tumor cells treated with EX527 under glucose deficiency or oxidative stress exhibited higher apoptosis rates than the control groups (Figure 2D). We then conducted a CCK-8 assay to establish the effects of SIRT1 on tumor cell proliferation under stress conditions. Both glucose deprivation and oxidative stress severely hindered proliferation in SIRT1-inhibited malignant cells but only moderately impaired growth in the control groups (Figure 2E). The foregoing results suggest that tumor cells induced SIRT1 under stress conditions to maintain survival and proliferation.

### SIRT1 might participate in the reprogramming of glucolipid metabolism in colorectal tumor cells

**Figure 3:**
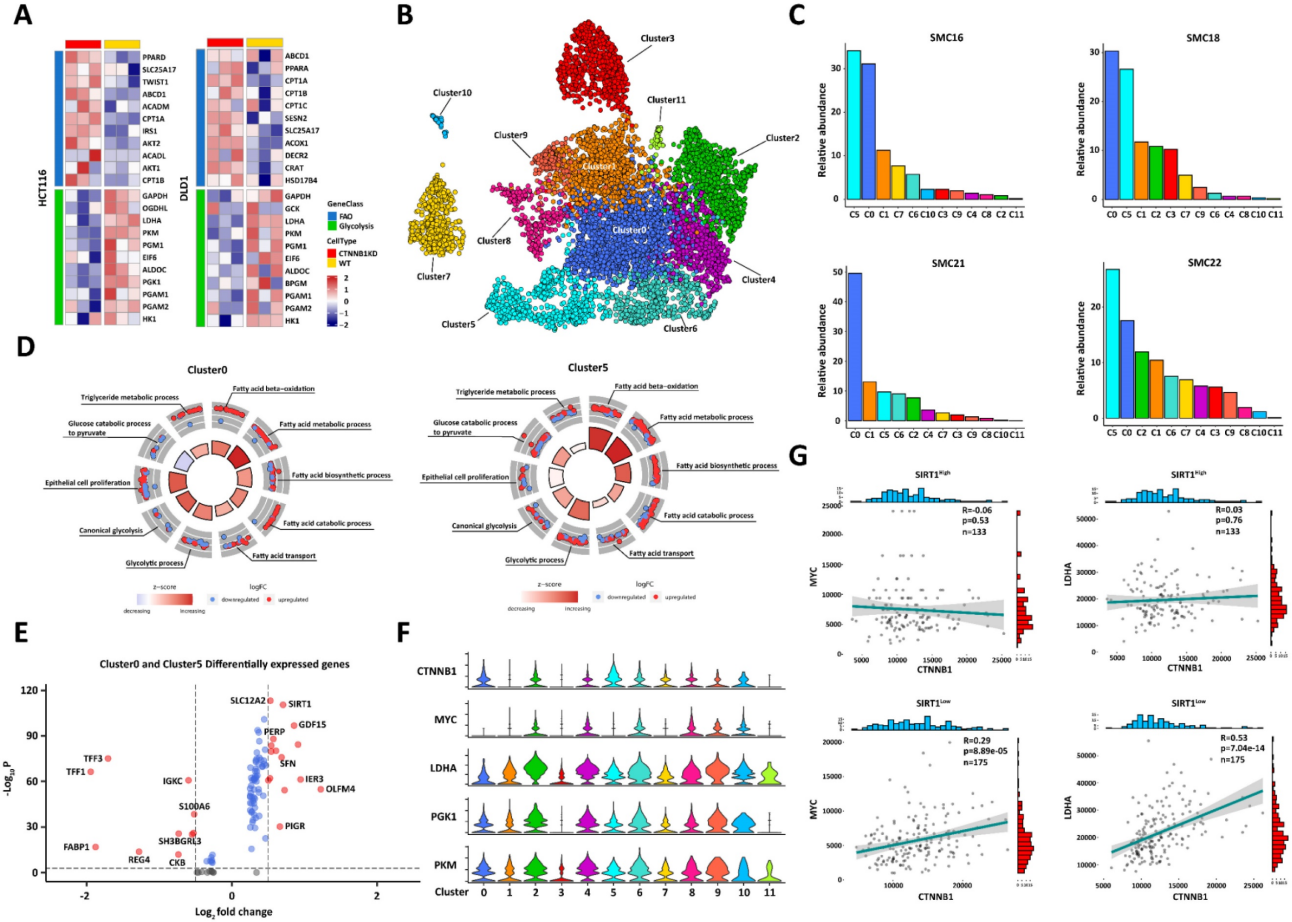
** SIRT1 may help reprogram glucolipid metabolism in colorectal tumor cells. (A)** Heatmap of fatty acid β-oxidation- and glycolysis-related genes in comparison between control and CTNNB1 KD HCT116 and DLD1 cells. **(B)** Single-cell RNA sequencing data for 23 Korean patients were obtained from GEO database (No. GSE132465) and integrated with Harmony v. 0.1.0. Cell types were annotated with SingleR v. 1.8.0. UMAP plot of all 7,186 epithelial (tumor) cells showing 12 clusters. **(C)** Relative abundances of all 12 tumor cell clusters in different patients. **(D)** GSEA of genes in cluster0 and cluster5. The expression level of each gene was normalized by row to z-score. Colors of inside sectors reflect relative enrichment levels of corresponding pathways across all tumor subclusters. Red and blue dots represent upregulation and downregulation, respectively. Pathways shown have P < 0.05. Inside sector height increases with decreasing p-value. **(E)** Volcano plot of RNA expression in cluster0 and cluster5 compared with other groups. Genes in upper left and right quadrants are significantly differentially expressed. **(F)** Violin plot showing expression patterns of glycolysis-related genes in 12 tumor cell clusters. **(G)** We divided 308 different colorectal tumor samples from the TCGA database into two groups according to mean SIRT1 expression. We calculated the correlations between MYC-CTNNB1 and LDHA-CTNNB1 in the high- and low-SIRT1 expression groups.

β-catenin plays a vital role in glycolysis. We conducted RNA sequencing on CRC cells presenting with β-catenin downregulation to predict metabolic conversion in tumor cells with poor glycolytic activity. The CTNNB1 (coding gene of β-catenin) KD CRC cells displayed upregulated FAO-related genes, whereas the WT tumor cells exhibited enhanced glycolytic capacity. Thus, oxidative FA catabolism is an important alternative energy supply in glycolytic crises (Figure 3A). We utilized published single-cell RNA sequencing data for 23 Korean CRC patients (No. GSE132465) to elucidate the mechanisms by which glucolipid metabolism is reprogrammed in tumor development 23. We applied a principal component analysis (PCA) to differentially expressed genes (DEGs) in all cell types and identified 12 tumor cell clusters (Figure 3B). The tumor microenvironment (TME) has an uneven nutrient supply. Therefore, only tumor cells with superior metabolic adaptability can achieve rapid *in vivo* proliferation. Patients SMC16, SMC18, SMC21, and SMC22, had higher tumor cell proportions (> 75% of all total tissue cells) than the others. We analyzed the compositions of the subclusters in these patients and found that their cluster0 and cluster5 ratios were higher than those in the other groups (Figure 3C). We then performed a gene set enrichment analysis (GSEA) on all tumor cell clusters and found that cluster0 and cluster5 exhibited enhanced FAO, glycolysis, and epithelial proliferation. Hence, they presented with a flexible capacity to reprogram glucolipid metabolism (Figure 3D). We compared the DEGs among groups and detected significantly upregulated SIRT1 and GDF15 24) in cluster0 and cluster5 (Figure 3E). GDF15 is a key FAO regulator.

We then evaluated the glycolytic capacity of various tumor cell subclusters. Cluster0 and cluster5 could not translate the highest β-catenin level to the upregulation of the key glycolysis-related genes *MYC, LDHA, PGK1,* and* PKM* (Figure 3F). We then hypothesized that *SIRT1* regulates the transcriptional function of β-catenin. To test this theory, we divided CRC samples from the TCGA database into strongly expressing (n = 133) and weakly expressing (n = 175) groups by calculating their average SIRT1 expression levels. β-catenin expression was positively correlated with those of *MYC* and *LDHA* in the SIRT1^Low^ groups but not in the SIRT1^High^ groups. Thus, SIRT1 negatively regulated the pro-glycolytic function of β-catenin (Figure 3G). Collectively, the foregoing bioinformatic analyses indicated that SIRT1 might be implicated in the metabolic conversion of glucolipids and augment the metabolic flexibility of tumor cells by promoting FAO while inhibiting glycolysis.

### SIRT1 deacetylates and promotes the cytoplasmic translocation of β-catenin

**Figure 4:**
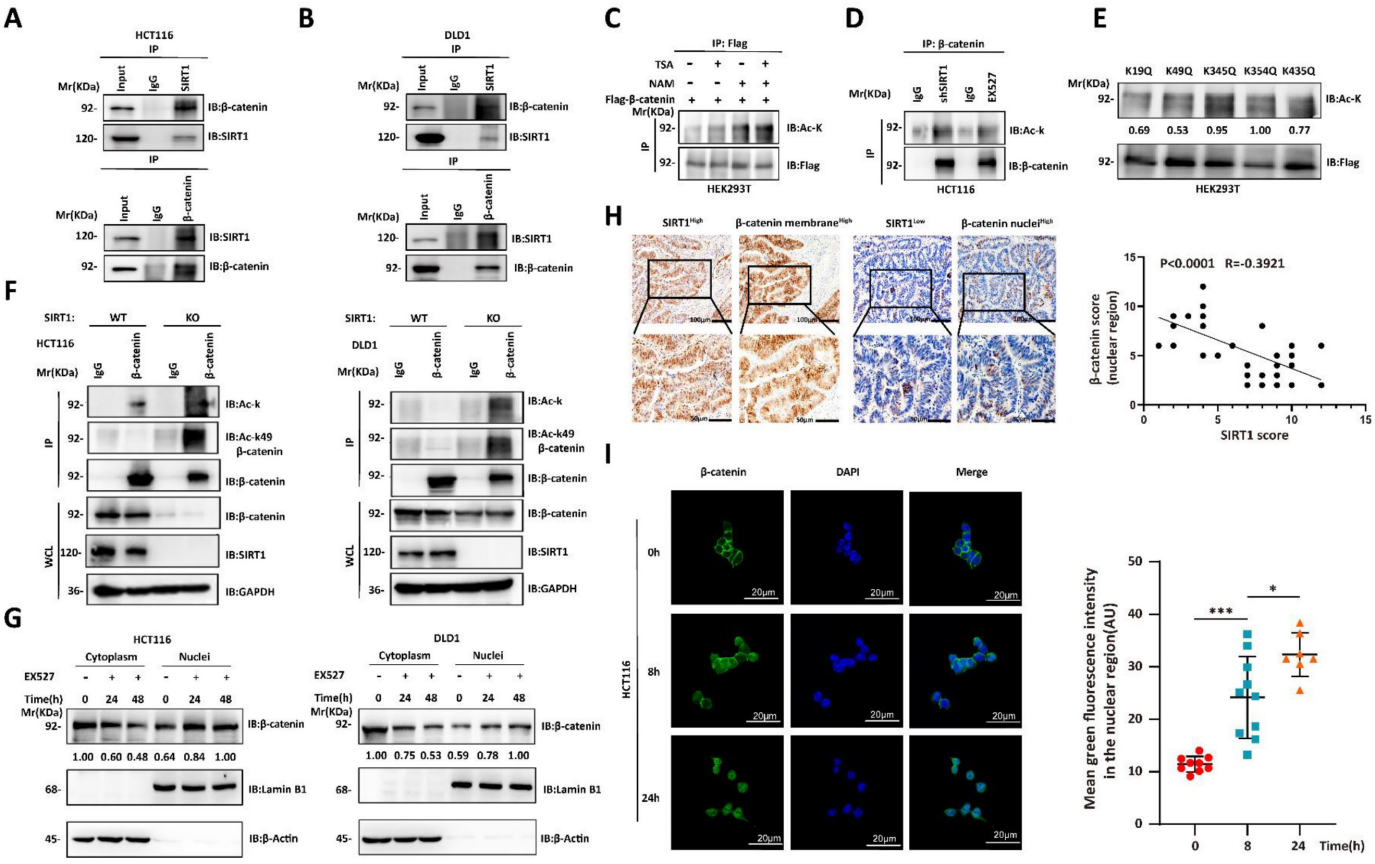
** SIRT1 deacetylates and promotes cytoplasmic translocation of β-catenin. (A-B)** Co-immunoprecipitation assay detecting SIRT1-β-catenin binding in HCT116 and DLD1 cells. Cell lysates were immunoprecipitated with anti-SIRT1 or IgG control and immunoblotted with anti-SIRT1 or anti-β-catenin. **(C)** Flag-β-catenin was transfected into HEK293T cells, which were then subjected to the deacetylase inhibitors TSA (500 nM) or NAM (5 mM). β-catenin acetylation and protein levels were analyzed by western blotting using the indicated antibody. **(D)** β-catenin acetylation was measured by western blotting and pan-acetylation antibodies in HCT116 cells treated with shSIRT1 or 20 μM EX527. **(E)** Indicated plasmids were transfected into HEK293T cells, and mutant acetylation was measured by western blotting. **(F)** β-catenin acetylation was measured by western blotting, pan-acetylation, and K49 site-specific acetylation antibody in SIRT1 KO and control tumor cells.** (G)** HCT116 and DLD1 cells treated with EX527 were harvested at 0 h, 24 h, and 48 h, and the cytosolic and nuclear fractions were separated. β-catenin expression in different fractions was analyzed by western blotting. **(H)** Representative photomicrographs showing subcellular β-catenin location in clinical colorectal carcinoma samples with high and low SIRT1 expression. Scale bars = 100 μm (upper panel) and 50 μm (lower panel). Correlations between nuclear β-catenin and SIRT1 among specimens were statistically significant (R = -0.3921; P < 0.0001; Pearson's correlation). **(I)** Confocal microscopy was used to examine subcellular β-catenin localization in HCT116 cells subjected to 20 μM EX527 for 0 h, 8 h, and 24 h. Scale bar = 20 μm; n = 2; technical replicates. Green fluorescence intensities represent β-catenin densities in nuclei at various time points and were determined with ImageJ. *P < 0.05, ***P < 0.001, ns: nonsignificant.

We then investigated the mechanisms by which SIRT1 regulates the biological function of β-catenin. The Wnt/β-catenin signaling pathway plays crucial role in tumorigenesis. The interactions between SIRT1 and the components of the Wnt/β-catenin pathway may promote tumor development 25, 26. We used co-immunoprecipitation to verify endogenous SIRT1-β-catenin interactions in CRC cell lines (Figures 4A-4B). As SIRT1 is a deacetylase, we evaluated its effects on β-catenin. β-catenin acetylation significantly increased in response to treatment with the SIRT inhibitor NAM, shSIRT1, and the SIRT1 inhibitor EX527 (Figures 4C-4D). We predicted the β-catenin acetylation sites using the PhosphoSitePlus P^TM^ database, individually mutated each of the putative acetylation sites to glutamine (Q), and examined their impact on acetylation. Glutamine substitution of K49 dramatically decreased β-catenin acetylation (Figure 4E). K49 acetylation was enhanced in SIRT1 KO HCT116 and DLD1 cells (Figure 4F). The foregoing data demonstrated that SIRT1 deacetylates β-catenin at K49. We also investigated whether the acetylation state affects subcellular β-catenin localization. SIRT1 interference promoted nuclear and inhibited cytoplasmic β-catenin accumulation (Figure 4G). We then validated nuclear-cytoplasmic β-catenin translocation by conducting IHC on clinical samples. We found that SIRT1 expression was negatively correlated with nuclear β-catenin (Figure 4H). We also found that nuclear β-catenin accumulation reached a maximum in colorectal tumor cells exposed to EX527 for 24 h (Figure 4I). Thus, SIRT1 deacetylated β-catenin at K49 and promoted its cytoplasm translocation.

### Deacetylation destroys the proglycolytic function of β-catenin

**Figure 5:**
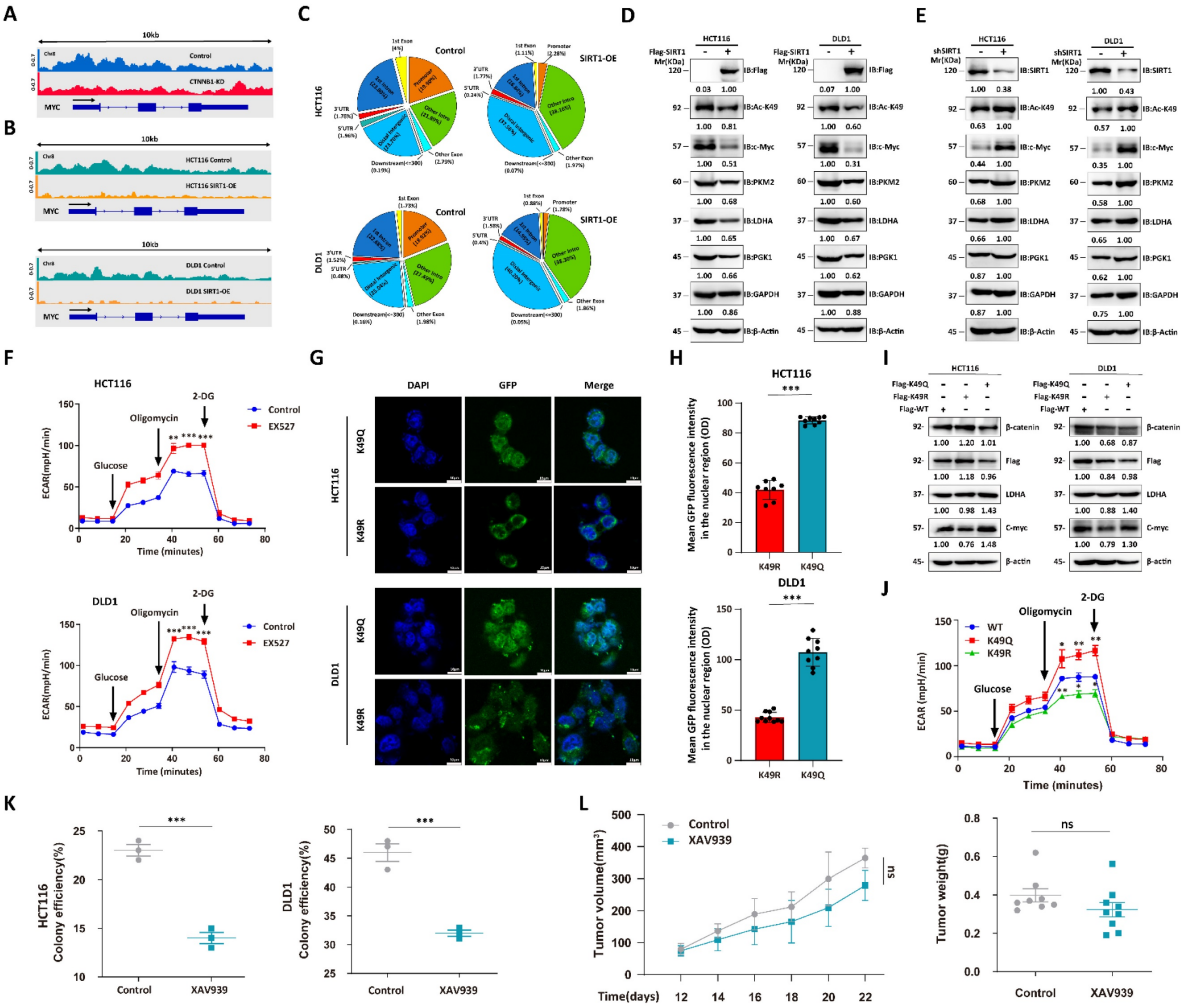
** Deacetylation destroys pro-glycolytic function of β-catenin. (A)** IGV snapshot of transcriptome analysis of *MYC* between HCT116-WT and HCT116-CTNNB1 KD cells. **(B)** IGV snapshot of transcriptome analysis of *MYC* between wild-type and SIRT1 overexpression tumor cells. **(C)** % of β-catenin interaction sites in genomes of HCT116 and DLD1 cells with SIRT1 overexpression. **(D)** Immunoblot analysis of glycolysis-related genes in malignant cells treated with control Flag vs. Flag-SIRT1. **(E)** Immunoblot analysis of glycolysis-related genes in malignant cells treated with control shRNA vs. SIRT1 shRNA. **(F)** ECAR was examined in HCT116 and DLD1 cells treated with 20 μM EX527. Each assay consisted of 3 wells each for the treatment and control groups. Data are means ± SEM of three independent assays. **P < 0.01, ***P < 0.001. **(G)** CTNNB1 KD HCT116 cells and DLD1 cells transfected with GFP-tagged CTNNB1 WT, mutant K49Q and mutant K49R. Then the subcellular localization of transfected β-catenin was examined by confocal microscope. Scale bar, 10μm. **(H)** The fluorescence intensity of GFP in nuclei of cells in **(G)** were determined using ImageJ. The number of cells used in statistics for each cell type ranged from 8-10. ***P < 0.001. **(I)** Analysis of key glycolytic genes expression in CTNNB1 KD HCT116 cells and DLD1 cells transfected with CTNNB1 WT, mutant K49Q and mutant K49R respectively. **(J)** HCT116 CTNNB1 KD were transfected with CTNNB1 WT, mutant K49Q and mutant K49R. ECAR were examined by Seahorse XFp extracellular flux analyzer. Each assay consisted of 3 wells each for the treatment and control groups. Data are means ± SEM of three independent assays. WT-CTNNB1 was regarded as reference. *P < 0.05, **P < 0.01, ***P < 0.001. **(K)** Colony-forming efficiencies were determined for HCT116 and DLD1 cells treated with 10 μM XAV939. ***P < 0.001. **(L)** The tumor suppression effect of 10 mg/kg XAV939 was evaluated in nude mice subcutaneously injected with DLD1 cells (1 × 10^6^/mouse, n = 8/group). From day 12 after the initial injection, tumor volumes were measured with a vernier caliper every 3 d. Tumor tissues were collected and weighed at the end of the assay. ***P < 0.001, ns: nonsignificant.

We performed chromatin immunoprecipitation coupled with ultra-high-throughput DNA sequencing (ChIP-Seq) to determine the roles of acetylation status in regulating *β-catenin* expression. The c-Myc regulates glucose metabolism 27. We used pan-acetylation antibodies to capture the gene segments and observed that CTNNB1 KD attenuated the peaks around the MYC promoter region (Figure 5A). As SIRT1 regulates subcellular β-catenin localization, we conducted ChIP-Seq on Flag-tagged, β-catenin-transfected CRC cell lines overexpressing SIRT1 and captured the gene segments with Flag antibody. SIRT1 overexpression lowered the peaks surrounding the MYC promoter region (Figure 5B). The < 1 kb transcription start site (TSS) distribution of the Flag-tag peaks was greater in the SIRT1-WT than in the SIRT1-OE cells. For this reason, deacetylation attenuated the transcriptional function of β-catenin (Figure 5C). We validated the function of acetylated β-catenin in CRC cell lines by overexpressing Flag-tagged SIRT1 and detected downregulation of the glycolytic proteins (Figure 5D). CRC cells treated with short hairpin SIRT1 (shSIRT1) presented with elevated K49 acetylation in β-catenin and glycolytic-related protein upregulation (Figure 5E). The EX527 treatment enhanced the extracellular acidification rate (ECAR) in malignant cells (Figure 5F). Moreover, we simulated different β-catenin acetylation state by transfecting CTNNB1 KD tumor cells with CTNNB1-WT, mutant K49Q (acetylation-mimetic) and K49R (deacetylation-mimetic) to compare their effects on cellular metabolism. Correspondingly, immunofluorescence results indicated that K49Q mutants gathered in nuclei while K49R enriched in cytoplasm (Figure 5G-H). Tumor cells transfected with K49Q mutant exhibited greater key glycolytic genes expression and ECAR compared with K49R mutant (Figure 5I-5J). We also evaluated the effects of β-catenin on tumor cells under different conditions. In the presence of sufficient glucose supply, tumor cell growth and colony formation were significantly suppressed in response to the β-catenin inhibitor XAV939 (Figure 5K). Under glucose deficiency, however (*in vivo* assay), XAV939 had moderate antitumor efficacy (Figure 5L). The foregoing findings suggest that inhibition of glycolysis had only a limited impact on tumor development. They also demonstrated that deacetylation attenuates the proglycolytic capacity of β-catenin.

### SIRT1 is positively correlated with the FAO capacity of CRC cells

**Figure 6:**
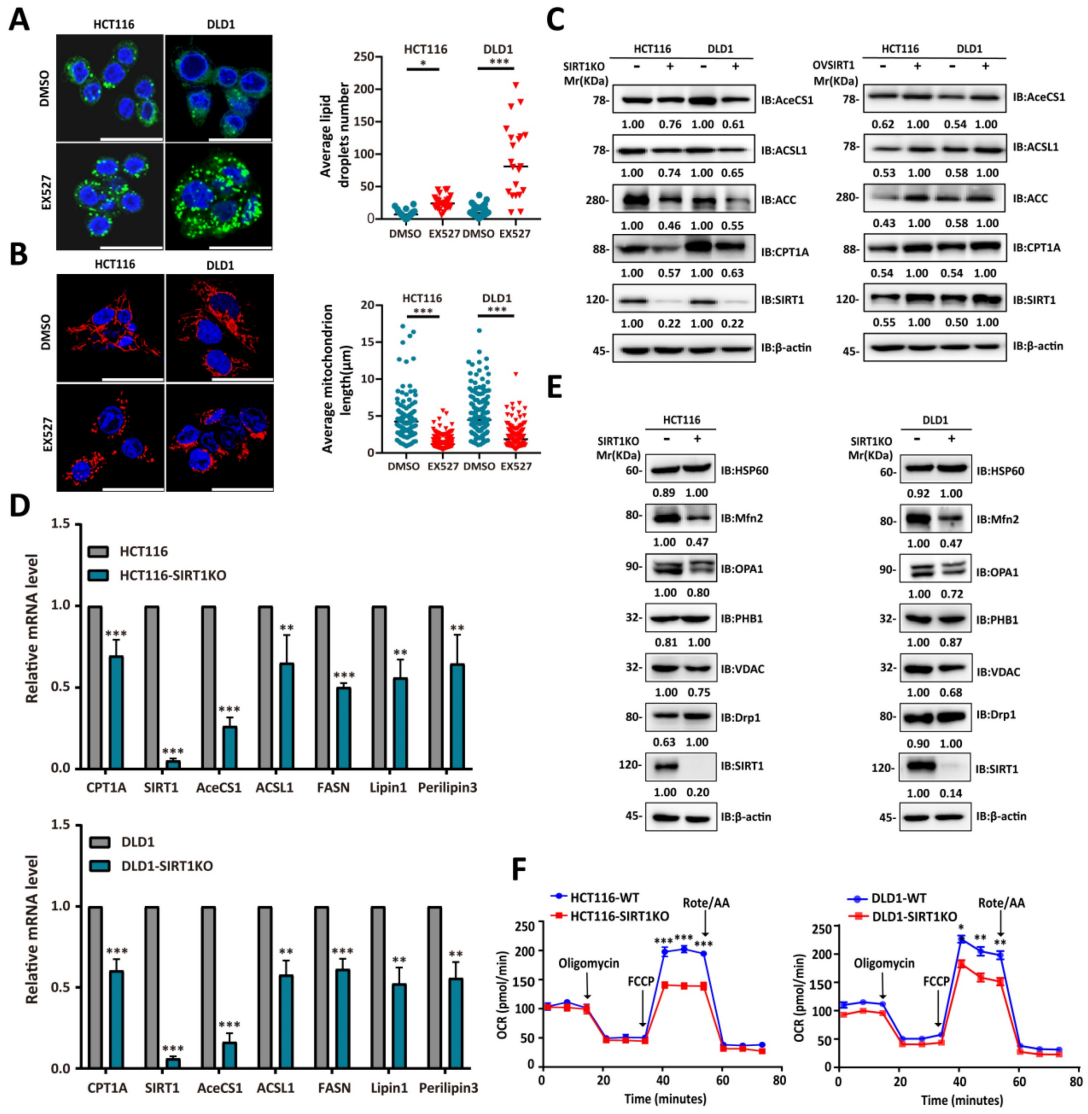
** SIRT1 expression was positively correlated with the FAO capacity of CRC cells. (A)** Confocal microscopy was used to observe lipid droplets in HCT116 and DLD1 cells treated either with DMSO or 20 μM EX527 for 24 h. Lipid droplets were stained with BODIPY (green). Nuclei were counterstained with Nucblue (blue). Scale bar = 25 μm. Representative of two experiments. Mean numbers of lipid droplets in different cells (n = 20/group) were determined with ImageJ. *P < 0.05, ***P < 0.001. **(B)** Confocal microscopy was used to observe mitochondrial morphology in HCT116 and DLD1 cells treated either with DMSO or 20 μM EX527 for 24 h. Mitochondria were stained with Mito-tracker (red). Nuclei were counterstained with Nucblue (blue). Scale bar = 25 μm. Representative of two experiments. Mean lengths of mitochondria in different cells (n = 20/group) were determined with ImageJ. ***P < 0.001. **(C)** Immunoblot analysis of protein extracts of colorectal tumor cells with SIRT1 KO or SIRT1 overexpression and probed for key lipid catabolism enzymes. β-actin was the loading control. **(D)** Expression levels of genes encoding key lipid catabolism enzymes in control and SIRT1 KO colorectal tumor cells were determined by RT-qPCR. **P < 0.01, ***P < 0.001. **(E)** Expression levels of mitochondrial fusion- and fission-related proteins in control and SIRT1 KO colorectal tumor cells were determined by western-blot. β-actin was the loading control. **(F)** OCR of control and SIRT1 KO colorectal tumor cells were determined with a Seahorse XFp extracellular flux analyzer. Data are means ± SD of three independent experiments. Each assay consisted of two wells each for the treatment and control groups. *P < 0.05, **P < 0.01, ***P < 0.001.

The single-cell RNA sequencing data and gene expression profiling interactive analysis (GEPIA; gepia.cancer-pku.cn) suggested that SIRT1 is upregulated under stress conditions to promote FAO. Downregulation of the latter results in cytoplasmic lipid droplet accumulation as a consequence of diminished FA utilization 28. To determine whether SIRT1 and FAO expression are closely associated, we exposed CRC cells to the SIRT1 inhibitor EX527 and observed adiposome accumulation (Figure 6A). Mitochondrial function and morphology are closely related. Fused mitochondria have relatively higher oxidative respiration capacity 29. The mitochondrion is the main site of FAO. SIRT1 downregulation in the tumor cells caused them to present with punctate mitochondria and diminished functionality (Figure 6B). We observed a positive correlation between SIRT1 and the lipid-metabolizing enzymes CPT1A (carnitine palmitoyltransferase 1A), AceCS1 (acetyl coenzyme A synthetase-1), and ACSL1 (acyl-CoA synthetase long-chain family member 1) at both the mRNA and protein levels (Figures 6C-6D). We also examined the protein expression levels of critical regulators of mitochondrial dynamics. We discovered downregulation of mito-fusion protein OPA1 (mitochondrial dynamin-like 120-kDa protein) and MFN2 (mitofusin-2) and upregulation of mito-fission protein DRP1 (dynamin-related protein 1) in tumor cells with SIRT1 KO (Figure 6E). We examined the OCR reflecting the tumor cell FAO capacity. OCR was markedly decreased in SIRT1 KO colon cancer cells. Hence, SIRT1 downregulation attenuated FAO (Figure 6F). The foregoing data demonstrated that SIRT1 expression is positively correlated with FAO activity in CRC cells.

### SIRT1 predicts rapid tumor progression and is a potential therapeutic target

We previously demonstrated that SIRT1 was upregulated in colorectal tumor cells deficient in glucose to promote the conversion of alternate energy sources. Glycolysis power cancer cells, and their rapid proliferation creates a glucose supply shock in the TME 30. We speculated that the SIRT1 level is promising as a tumor progression biomarker and a therapeutic target.

**Figure 7:**
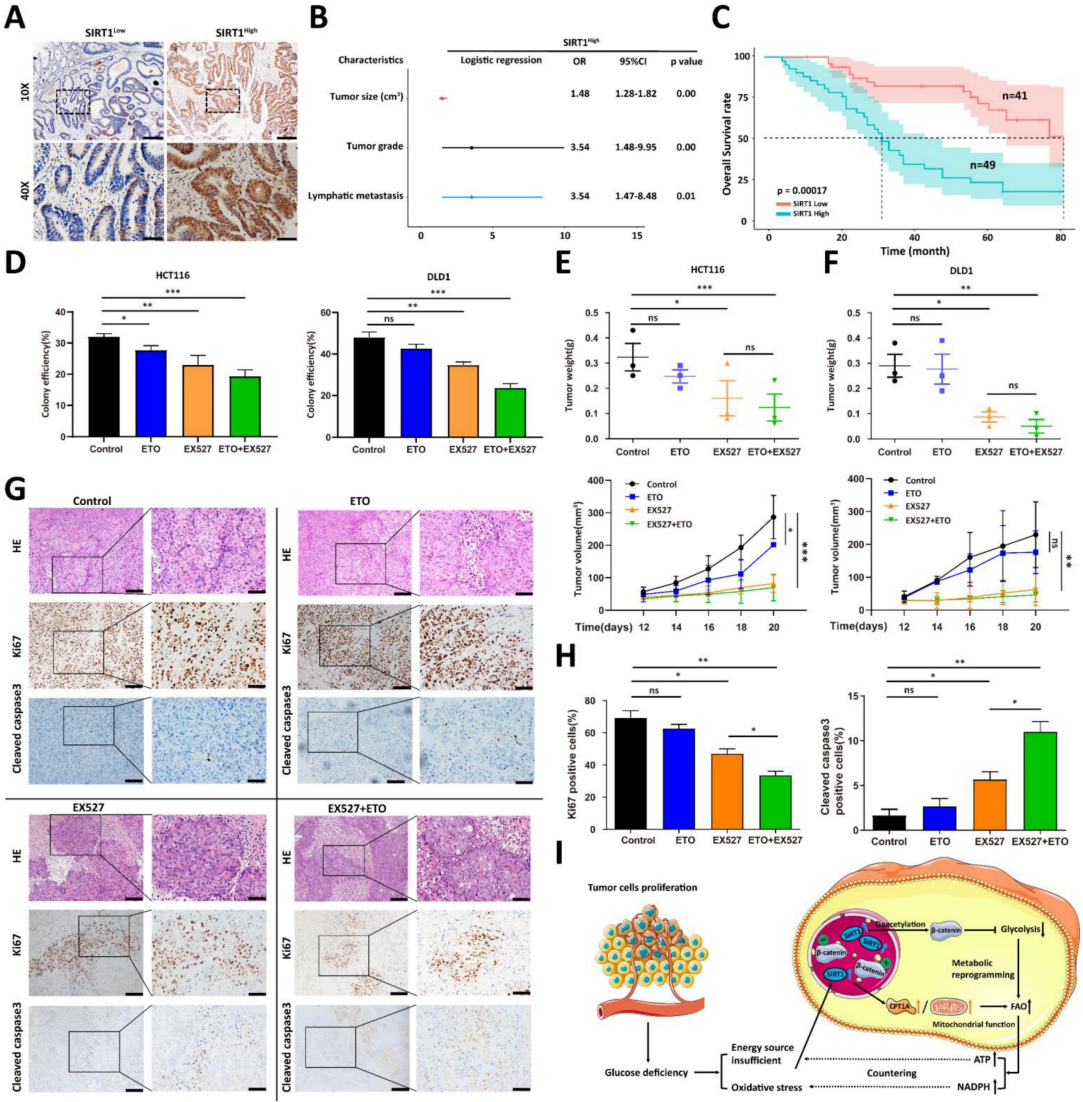
** SIRT1 predicts rapid tumor progression and is a potential therapeutic target. (A)** Representative IHC staining for SIRT1 in a tissue microarray of patients with colorectal cancer. Based on mean IHC staining scores for SIRT1, the clinical samples were divided into high SIRT1 expression (SIRT1^High^ n = 49) and low SIRT1 expression (SIRT1^Low^ n = 41) groups. Scale bars = 200 μm (upper panel) and 50 μm (lower panel). **(B)** Multivariate logistic regression analysis of odds ratios (ORs) for various clinicopathological characteristics. **(C)** Kaplan-Meier survival analysis of associations between SIRT1 expression levels and OS rates in patients with CRC. Red line: SIRT1^Low^. Blue line: SIRT1^High^. **(D)** Colony-forming efficiency was determined for HCT116 and DLD1 cells treated with 20 μM EX527 or 100 μM ETO alone or in combination. (E-F) Tumor suppression effects of 30 mg/kg EX527 or 40 mg/kg ETO alone or in combination were evaluated in nude mice subcutaneously injected with HCT116 or DLD1 cells (1 × 10^6^/mouse, n = 3/group). From day 12 after the initial injection, tumor volumes were measured with a vernier caliper every 3 d. Tumor tissues were collected and weighed at the end of the assay. *P < 0.05, **P < 0.01, ***P < 0.001, ns: nonsignificant. **(G)** Representative IHC staining of H&E, Ki-67, and cleaved caspase3 in tumor tissues collected at the end of the assay (DLD1). Scale bars = 100 μm (left panel) and 50 μm (right panel). **(H)** Statistical analysis of IHC-determined Ki-67 and cleaved caspase3 expression in tumor tissues (DLD1). *P < 0.05, **P < 0.01, ***P < 0.001, ns: nonsignificant. **(I)** Schematic illustration of colorectal carcinoma cells responding to glucose deficiency and oxidative stress via SIRT1 upregulation and consequent induction of glucolipid metabolism.

We analyzed the clinical relevance of SIRT1 expression in human CRC samples to validate the preceding hypothesis. We divided 90 human CRC samples into two groups (SIRT1^High^ and SIRT1^Low^) based on their mean SIRT1 expression levels (Figure 7A). SIRT1 upregulation was significantly associated with several aggressive clinicopathological features of CRC, such as large tumor size and high recurrence and metastasis rates (Figure 7B). A Kaplan-Meier survival analysis indicated that SIRT1^High^ profile patients had a worse prognosis than those with the SIRT1^Low^ phenotype (Figure 7C). We previously demonstrated that the inhibition of glycolysis *per se* had only a limited impact on *in vivo* tumor development (Figure 5H). We evaluated the tumor suppression efficacies of EX527 and the FAO-specific inhibitor etomoxir (ETO) alone and in combination. The aim was to validate the importance of the transformation of glucolipid metabolism in tumor development and the therapeutic potential of SIRT1 interference in CRC patients. Colony formation and *in vivo* assays revealed that ETO elicited a weak response, whereas EX527 alone or in combination with ETO significantly inhibited tumor growth (Figures 7D-7F). IHC assays of tumor tissue collected at the endpoints of the mouse xenograft trials showed that the group administered the EX527-ETO combination presented with the lowest and highest expression levels of the cell proliferation marker Ki-67 and the apoptosis marker cleaved caspase3, respectively. Thus, tumor development was constrained (Figures 7G-7H).

The foregoing results demonstrated that FAO inhibition alone does not significantly restrict tumor growth, but interference with glycolipid metabolism conversion might have superior therapeutic efficacy. Our data suggested that SIRT1 plays a crucial role in the reprogramming of colorectal tumor cell metabolism and is an effective prognostic tool and a promising therapeutic target.

## Discussion

Metabolic reprogramming in extreme and complex tumor microenvironments (TMEs) is regarded as a major contributor of tumor initiation, growth, and metastatic dissemination in colorectal cancers (CRC) 31. Despite tumor cell heterogeneity caused by various genetic and environmental factors, the cellular metabolic pathways nonetheless follow certain consistent rules. These patterns confer vulnerabilities on the tumors and facilitate their therapeutic targeting 32. In the present study, we identified sirtuin-1 (SIRT1) as the hub of the transformation of glucolipid metabolism in colorectal tumor cells (Figure 7I). Under glucose deficiency, SIRT1 upregulation impaired the transcriptional activity of β-catenin via deacetylation, attenuated glycolysis, reduced the glucose requirement, and promoted fatty acid (FA) oxidation by activating key lipid metabolism enzymes. Thus, SIRT1 expression is a promising indicator of the conversion frequency of glucolipid metabolism and a predictor of tumor progression in CRC.

Aerobic glycolysis is the dominant energy supply route in tumor cells as it rapidly generates ATP and provides macromolecular metabolites for biosynthesis. Nevertheless, the Warburg effect inefficiently supplies energy, consumes copious amounts of glucose, produces relatively little ATP, causes glucose deprivation, and induces metabolic stress during cancer development 33. Glucose deprivation impairs glycolysis and the pentose phosphate pathway (PPP), thereby eliciting oxidative stress and causing redox imbalance and cell death 22. Increases in glutaminolysis and *de novo* fatty acid biosynthesis are also prominent hallmarks of cancer 15. Lipid metabolism and especially fatty acid oxidation (FAO) generate abundant ATP by using fatty acids. They also participate in NADPH production to neutralize oxidative stress. Thus, glycolysis and FAO complement each other and play vital roles in tumor progression. However, the mechanisms by which glucolipid metabolism is reprogrammed are unknown. Recent work has shown that the PI3K-LDHA positive feedback circuit mechanistically explains the Warburg effect, and LDHA is widely regarded as a desirable therapeutic target for cancers 4, 5. Carnitine palmitoyltransferase1A (CPT1A) catalyzes the rate-limiting step in FAO, activates FAO, and fuels cancer growth via ATP and NADPH production 34. We speculated that the biomolecule regulating both LDHA and CPT1A is the pivot of both glycolysis and the transformation of FAO metabolism.

The Wnt/β-catenin pathway transcriptionally regulates glycolysis-related genes 7. To perform these functions, however, β-catenin must first be localized to the nucleus. We discovered that the acetylation status of β-catenin that determines its subcellular localization. K49 acetylates β-catenin enriched in the nuclei so that it can upregulate the glycolysis-related genes *LDHA, GLUT1,* and* PKM2*. Deacetylated β-catenin is translocated to the cytoplasm or cell membrane, and glycolysis is attenuated. Deacetylase SIRT1 regulates β-catenin. SIRT1 is a vital energy status sensor, protects cells against metabolic stress, and is a promising target for aging-related diseases 35. SIRT1 protein levels are elevated in inflammation and oxidative stress 36. We induced SIRT1 upregulation in a CRC cell line by mimicking glucose deprivation via glucose deficiency and exogenous H_2_O_2_. We also demonstrated a positive correlation between SIRT1 and CPT1A and found that SIRT1 upregulation activated FAO. Therefore, CRC cells upregulate SIRT1, enhance FAO, attenuate glycolysis, and reprogram glucolipid metabolism under inadequate glucose supply.

The present study disclosed the clinical relevance of SIRT1. During tumor development, malignant cells are often subjected to stressors such as glucose deprivation. Thus, SIRT1 expression is no longer regarded as an intrinsic characterization of malignant cells. Rather, it is now considered an indicator of the frequency of glucolipid transformation and rapid tumor progression. SIRT1 plays pivotal roles in metabolic reprogramming between glycolysis and FAO and is, therefore, a potential therapeutic target restricting tumor growth. Honey is recognized as a promising natural food with anticancer efficacy as it induces ROS accumulation and impairs the Wnt/β-catenin pathway in CRC cells. These conditions resemble the glucose deprivation that occurs during tumor development 37. However, honey also contains the compound chrysin, which activates SIRT1 38. Thus, administering honey with low chrysin content or in combination with a SIRT1 inhibitor would be preferable in CRC treatment. Moreover, severe immune deficit usually accompanies rapid tumor progression. Future investigations should endeavor to determine whether SIRT1 upregulation causes immunosuppression in the TME.

## Materials and Methods

### Cell lines and culture

HEK293T and human CRC cell lines (HCT116 and DLD1) were purchased from ATCC (American Type Culture Collection, Manassas, VA, USA). The HCT116 and HEK293T cells were cultured in Dulbecco's modified Eagle's medium (DMEM). The DLD1 cells were cultured in Roswell Park Memorial Institute (RPMI) 1640 medium. All media were supplemented with 10% (v/v) fetal bovine serum (FBS), 100 U penicillin, and 100 μg mL^-1^ streptomycin. All cell lines were cultured in a humidified 5% CO_2_ atmosphere at 37 ℃.

### Plasmids, short hairpin (sh) RNAs, and transfection

SIRT1 and CRISPR/Cas9 KO plasmids (No. sc-400085; Santa Cruz Biotechnology, Dallas, TX, USA) and shSIRT1 were transfected into the cells with Lipofectamine^TM^ 3000 (No. L3000015; Invitrogen, Carlsbad, CA, USA) following the manufacturers' instructions. Cells at 30% confluence were incubated in media containing optimal lentivirus dilutions mixed with polybrene. After 48 h transfection, the cells were subjected to 5 mg/mL puromycin selection to obtain stable transfected cells. The oligonucleotide sequences of the shRNAs were as follows: scrambled shRNA: 5′-CGTACGCGGAATACTTCGA-3′; *SIRT1* shRNA: 5′-GCGGGAATC-CAAAGGATAATT-3′; and β-catenin shRNA: 5′-GGATGTGGATACCTCCCAA-3′.

### OCR and ECAR assays

Extracellular acidification rates (ECAR) and cellular oxygen consumption rates (OCR) were measured with a Seahorse XFp extracellular flux analyzer (XF HS Mini; Agilent Technologies, Santa Clara, CA, USA) according to the manufacturer's instructions. ECAR and OCR were measured with a Seahorse XFp Glycolysis Stress Test Kit (No. 103017-100; Agilent Technologies) and a Seahorse XFp Cell Mito Stress Test Kit (No. 103010-100; Agilent Technologies), respectively. Fifteen thousand cells per well were seeded into a Seahorse XFp cell culture microplate (Agilent Technologies) for 12 h. After baseline measurements, ECAR, 10 mM glucose, 1 μM oligomycin, and 50 mM of 2 deoxy-*D-*glucose (2-DG) were sequentially injected into each well at the indicated time points. For OCR, 2.5 μM oligomycin, 0.5 μM FCCP:carbonyl cyanide-4-(trifluoromethoxy)phenylhydrazone, and 0.5 μM antimycin A were sequentially injected. The data were analyzed with Seahorse XFp Wave software (Agilent Technologies). The OCR and ECAR units were pmols min^-1^ and mpH min^-1^, respectively.

### Western blotting and co-immunoprecipitation (Co-IP)

Whole-cell lysates were prepared with cell lysis buffer (No. 78501; Thermo Fisher Scientific, Waltham, MA, USA) containing a protease inhibitor cocktail (Roche Diagnostics, Basel, Switzerland). An equal amount of protein was subjected to sodium dodecyl sulfate-polyacrylamide gel electrophoresis (SDS-PAGE) and transferred onto nitrocellulose (NC) membranes (Millipore EMD, Billerica, MA, USA). The membranes were blocked with 5% (w/v) skim milk in phosphate-buffered saline-Tris (PBST) at RT for 2 h and incubated with primary antibodies overnight at 4 ℃ to detect target proteins. They were then incubated with secondary antibodies at RT for 1 h and the blots were visualized in a ChemiDoc Imaging System (Bio-Rad Laboratories, Hercules, CA, USA). For the co-IP, 100 µL cell lysate (No. #87788; Thermo Fisher Scientific) was mixed with 2 µL primary antibody at 4 ℃ for 3 h and pulled down with Protein A/G Magnetic Beads (No. 88803; Thermo Fisher Scientific) at 4 ℃ overnight. The IP beads were then washed thrice with lysis buffer and heated in SDS loading buffer at 100 ℃ for 10 min in preparation for SDS-PAGE and immunoblot analysis.

### RNA extraction and qRT-PCR

Total cellular RNA was extracted with a Total RNA Kit (No. R6834; Omega Bio-tek, Norcross, GA, USA) and reverse-transcribed into cDNA with a PrimeScriptRT Reagent Kit (No. RR047A; TaKaRa Bio Inc., Kusatsu, Shiga, Japan) and random primers according to the manufacturer's guidelines. The cDNA product was used as a template for target gene transcript amplification by real-time polymerase chain reaction (RT-PCR) with TB Green Premix Ex TaqⅡ (No. RR820A; TaKaRa Bio Inc.) on a LightCycler 480 Ⅱ Sequence Detection System (Roche Diagnostics). β-actin was the normalization control.

### Immunohistochemistry (IHC)

Cancer tissue specimens were fixed in 10% (v/v) formalin-PBS at room temperature (RT) for 48 h, transferred to an automatic tissue-dehydrating machine (HistoCore PEARL; Leica Microsystems, Wetzlar, Germany), and embedded in paraffin. The specimens were cut by microtome into sections 2-5 µm thick, deparaffinized, rehydrated, and blocked with 3% (v/v) H_2_O_2_ at 37 ℃ for 10 min. Tissue antigens were retrieved by heating the sections in a pressure cooker for 150 s. The sections were cooled to RT and incubated with primary antibodies against SIRT1 (No. ab110304; 1:500; Abcam, Cambridge, UK), β-catenin (No. 8480; 1:100; CST), Ki-67 (No. 9449; 1:1,000; CST), and cleaved caspase3 (No. 9661; 1:300; CST) at 4 ℃ overnight. The sections were then stained with an IHC detection kit (No. K5007; Agilent Technologies) according to the manufacturer's instructions.

### Immunofluorescence

Cells were fixed in 4% (v/v) paraformaldehyde (PFA) for 30 min, permeabilized with 0.5% (w/v) Triton X-100 for 15 min, blocked with 10% (v/v) goat serum for 1 h, washed with PBS, stained with primary antibodies at 4 ℃ overnight, washed again with PBS, and stained with secondary antibodies for 1 h. Nuclei were stained with Hoechst 33342 (No. H3570; Invitrogen). Images were captured by confocal microscopy (STELLARIS STED; Leica Microsystems).

### Lipid droplet and mitochondrial staining

BODIPY 493/503 dyes (No. D3922; Thermo Fisher Scientific) were used as they are effective lipid trafficking tracers and general-purpose membrane probes. The dyes were diluted to 3 µM with bovine serum albumin (BSA)-free, serum-free medium, and the cells were incubated in them in the dark for 30 min. MitoTracker Deep Red FM 644/665 (No. M22426; Thermo Fisher Scientific) is a far-IR fluorescent dye used for mitochondrial staining and localization in living cells. The dye was diluted to 100 nM with BSA-free medium, and the cells were incubated in it in the dark for 30 min. Nuclei were stained with NucBlue Live ReadyProbes (No. R37605; Invitrogen). The final cell density was 1 × 10^6^/mL. Images were captured by confocal microscopy (STELLARIS STED; Leica Microsystems).

### Single-cell RNA sequencing data analysis

A gene expression matrix was downloaded from the GEO database (https://www.ncbi.nlm.nih.gov/geo/) (No. GSE132465), normalized with the Seurat package (v. 4.0.6) in R (R Core Team, Vienna, Austria), and selected according to the criteria of the original article (PMID:32451460). The harmony package (v. 0.1.0) in R was used for batch correction. A principal component analysis (PCA) was performed using differentially expressed genes (DEGs), and resolutions of 0.5-1 were explored to optimize subcluster representation. The Seurat, PCA, and SingleR (v. 1.8.0 https://www.bioconductor.org/packages/release/bioc/html/SingleR.html) pipelines were combined for initial clustering and cell type identification.

### RNA-Seq, ChIP-Seq, and data analysis

Total RNA was isolated from normal and β-catenin KO tumor cells for RNA sequencing. RNA sequencing and raw data preprocessing were conducted by Shanghai Genergy Biotechnology Co. Ltd., Shanghai, China. Gene ontology (GO) enrichment analysis of the RNA-Seq gene expression matrix was conducted in the clusterProfiler v. 4.2.1 package in R. The heatmap was plotted with the ggplot2 v. 3.3.5 package in R. The ChIP assays were performed using a SimpleChIP Plus Sonication Chromatin IP Kit (No. 56383; CST) according to the manufacturer's protocols. Cells (4 × 10^6^) were fixed with 37% (v/v) formaldehyde to crosslink the DNA with the proteins. The chromatin was sheared and fragmented under the appropriate sonication conditions and incubated with antibodies and Protein G magnetic beads overnight. The reversed protein-DNA cross-link was purified, and DNA enrichment was detected for the sequence analysis. Sequencing and raw data preprocessing were performed by Beijing Novogene Co. Ltd., Beijing, China. Bed format files were used to annotate the peaks in the ChIPpeakAnno v. 3.28.0 and ChIPseeker v. 1.30.3 packages in R. Bw format files were used to conduct a transcriptome analysis in IGV v. 2.11.4.

### Human clinical samples

Colorectal tumor samples and patient clinical information (n = 90) were purchased from Shanghai Outdo Biotechnology Co. Ltd., Shanghai, China (Cat. No. HRec-Ade180Sur-04).

### Animal studies

All mouse experiments were performed according to the ethic permission (2019, No.177) of the Animal Care and Use Committees at the Medical Research Center of Chongqing University Cancer Hospital, Chongqing, China. For the tumor formation assay, the cells were prepared in PBS. CRC frequency was calculated by extreme limiting dilution analysis. Subcutaneous tumor models were used for the tumorigenic potential assays. In the subcutaneous tumor model, 1 × 10^6^ cells were subcutaneously injected into BALB/c nude mice. Tumor growth was measured with a vernier caliper every 2-3 d. Tumor volumes were calculated using the formula V = (a^2^ × b) / 2. At 26 d after cell implantation, the animals were euthanized, and their tumors were excised and weighed.

### Statistical analysis

Data were processed in GraphPad 7 (GraphPad Software, La Jolla, CA, USA) and R v. 4.1.2. All statistical analyses were performed using unpaired/paired *t*-tests or one-way analysis of variance (ANOVA). Kaplan-Meier survival curves and log-rank tests were used to estimate overall survival (OS) and differences among groups. A multivariate logistic regression model was run using the nnet v. 7.3-17 package in R to calculate the odds ratios (ORs) for various clinicopathological characteristics. Significance levels were set at *P < 0.05, **P < 0.01, and ***P < 0.001 for three independent experiments.

## Figures and Tables

**Table 1:** Antibodies used in the present study.

Protein target	Assay	Company and Product Code
Primary antibody		
SIRT1	WB (western blot)	Cell Signaling Technology (CST, Danvers, MA, USA) No. 9475
SIRT1	WB/immuno-histochemistry (IHC)/coimmuno-precipitation (Co-IP)	Santa Cruz Biotechnology No. sc-15404
β-Actin	WB	CST No. 3700
β-Catenin	WB/IHC/Co-IP	CST No. 8480
c-Myc	WB	CST No. 9402
GAPDH	WB	CST No. 5174
LaminB1	WB	CST No. 13435
Acetylated-lysine	WB	CST No. 9441
Acetyl-β-catenin (Lys49)	WB	CST No. 9030
PGK1	WB	CST No. 68540
LDHA	WB	CST No. 2012
PKM2	WB	CST No. 3198
CPT1A	WB	CST No. 12252
AceCS1	WB	CST No. 3658
ACSL1	WB	CST No. 9189
Acetyl-CoA Carboxylase	WB	CST No. 3662
DRP1	WB	CST No. 8570
VDAC	WB	CST No. 4661
PHB1	WB	CST No. 2426
OPA1	WB	BD Biosciences (Franklin Lakes, NJ, USA) No. 612606
MFN2	WB	Sigma-Aldrich Corp. (St. Louis, MO, USA) No. WH0009927M3
HSP60	WB	CST No. 12165
Cleaved caspase3	WB	CST No. 9664
Flag-tag	WB/Co-IP	CST No. 14793
Secondary antibody		
Goat anti-rabbit IgG	WB	Beijing Zhongshan Golden Bridge Biotechnology Co. Ltd., Beijing, China No. ZB-2301
Goat anti-mouse IgG	WB	Beijing Zhongshan Golden Bridge Biotechnology Co. Ltd. No. ZB-2305
Alexa Fluor 488 goat	Confocal	Thermo Fisher Scientific No. A-11008
anti-rabbit IgG		

**Table 2:** Primer sequences.

Gene	Forward primer	Reverse primer
CPT1A	ATCAATCGGACTCTGGAAACGG	TCAGGGAGTAGCGCATGGT
Perilipin 3	TATGCCTCCACCAAGGAGAG	ATTCGCTGGCTGATGCAATCT
FASN	AAGGACCTGTCTAGGTTTGATGC	TGGCTTCATAGGTGACTTCCA
ACSL1	CCATGAGCTGTTCCGGTATTT	CCGAAGCCCATAAGCGTGTT
Lipin 1	CCAGCCCAATGGAAACCTCC	AGGTGCATAGGGATAACTTCCTG
AceCS1	CACAGGACAGACAACAAGGTC	CCTGGGTATGGACGATGCC
SIRT1	TAGCCTTGTCAGATAAGGAAGGA	ACAGCTTCACAGTCAACTTTGT
β-Actin	CATGTACGTTGCTATCCAGGC	CTCCTTAATGTCACGCACGAT

## Abbreviations

acetyl coenzyme A synthetase-1): AceCS1
acyl-CoA synthetase long-chain family member 1): ACSL1
ChIP-seq: chromatin immunoprecipitation coupled with ultra-high-throughput DNA sequencing
CPT1A: carnitine palmitoyltransferase 1A
CRC: colorectal carcinoma
Dynamin-related protein 1): DRP1
ECAR: extracellular acidification rate
FAO: fatty acid oxidation
GEPIA: Gene Expression Profile Interactive Analysis
GSEA: gene set enrichment analysis
LDHA: lactate dehydrogenase A
Mitofusin-2): MFN2
NADPH: nicotinamide adenine dinucleotide phosphate
OCR: oxygen consumption rate
mitochondrial dynamin-like 120 kDa protein): OPA1
PCA: principal component analysis
ROS: reactive oxygen species
SIRT1: sirtuin-1
TSS: transcription start site
